# Resveratrol Improves Brain-Gut Axis by Regulation of 5-HT-Dependent Signaling in the Rat Model of Irritable Bowel Syndrome

**DOI:** 10.3389/fncel.2019.00030

**Published:** 2019-02-08

**Authors:** Ying-Cong Yu, Jing Li, Meixi Zhang, Jian-Chun Pan, Ying Yu, Jian-Bo Zhang, Liang Zheng, Jian-min Si, Ying Xu

**Affiliations:** ^1^Department of Gastroenterology, Wenzhou No. 3 Clinical Institute Affiliated to Wenzhou Medical University, Wenzhou People’s Hospital, Wenzhou, China; ^2^Institute of Gastroenterology, Zhejiang University, Hangzhou, China; ^3^Department of Pharmacy, The Affiliated Hospital of Qingdao University, Qingdao, China; ^4^Pingyang Hospital of Traditional Chinese Medicine, Pingyang, China; ^5^Brain Institute, School of Pharmacy, Wenzhou Medical University, Wenzhou, China; ^6^Department of Pharmaceutical Sciences, School of Pharmacy and Pharmaceutical Sciences, University at Buffalo, The State University of New York, Buffalo, NY, United States

**Keywords:** irritable bowel syndrome, chronic acute combining stress, pCREB, BDNF, 5-HT

## Abstract

Irritable bowel syndrome (IBS) is at high risk of co-morbid depression and anxiety, which reduces patients’ quality of life and increases the burden of health care costs. However, the pathophysiological mechanisms responsible for IBS still remain unknown. This study investigated the effects of resveratrol on stress-related depression, anxiety, intestinal and visceral dysfunction in rat model of IBS. Rats received chronic acute combining stress (CACS) for 22 days exhibited depression/anxiety-like behavior, visceral hypersensitivity and altered intestinal motility, as measured by the forced swimming, marble bury, abdominal withdrawal reflex (AWR) and intestinal tract motility (ITM) tests. These abnormalities were accompanied by reduced 5-hydroxytryptamine (5-HT) level in the hippocampus and increased 5-HT expression in the gut (ileum and colon) after CACS. Chronic treatment of IBS rats with resveratrol dose-dependently normalized CACS-induced both central nervous and peripheral dysfunction, which were consistent with its differentially regulating 5-HT contents in the brain and intestine. Pretreatment with the 5-HT_1A_ receptor antagonist NAN-190 hydrobromide (NAN-190) prevented such effects. While sub-threshold of 5-HT_1A_ receptor agonist 8-OH-DPAT potentiated the effects of low dose of resveratrol (10 mg/kg) on CACS-related behavioral abnormalities. Furthermore, resveratrol markedly increased PKA, p-cAMP-response element binding protein (p-CREB) and brain derived neurotrophic factor (BDNF) expression in the hippocampus of IBS rats, while decreased PKA, pCREB and BDNF levels were found in the ileum and colon. These effects were prevented by NAN-190, which were consistent with the behavioral changes. The present results suggested that resveratrol improved anti-IBS-like effects on depression, anxiety, visceral hypersensitivity and intestinal motility abnormality through regulating 5-HT_1A_-dependent PKA-CREB-BDNF signaling in the brain-gut axis.

## Introduction

**Figure 1:**
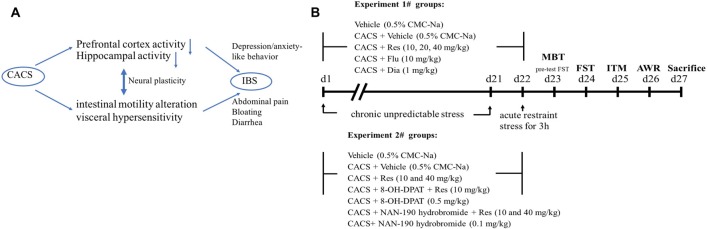
A schematic presentation about the link between brain and gut axis **(A)** and drug treatment and behavioral tests procedure **(B)**. Animals were treated with resveratrol, fluoxetine or diazepam, 1 h or 30 min before chronic acute combining stress (CACS) for 22 days. The behavioral tests were started from day 23 to 26. MBT, marble-burying task; FST, forced swimming test; ITM, intestinal tract motility; AWR, abdominal withdrawal reflex test.

Irritable bowel syndrome (IBS), a major form of functional gastrointestinal disease, is characterized by the abdominal pain and bloating, visceral hyperalgesia and bowel movements abnormalities, which affect approximately 11.2% of the world population (Enck et al., 2016). A high rate of psychological comorbidities is often seen in IBS patients besides their gastrointestinal disorders. For example, functional dyspepsia and gastroesophageal reflux disease comorbidity with anxiety, depression, somatization and neuroticism are prevalent in IBS patients (Kibune-Nagasako et al., 2016), which are likely to be affected by dysfunction of the brain-gut axis (Park et al., 2017). The role of the gut-brain axis is to monitor gut (ileal and colonic) functions and to link emotional centers of brain such as cortex and hippocampus with peripheral intestinal functions such as intestinal tract motility (ITM) and entero-endocrine signaling (Figure 1A). Recent studies indicated that peripheral hypersensitivity affects central nervous system (CNS), which may trigger depression or anxiety that in turn affects the visceral sensitivity and intestinal motility dysfunction (Enck et al., 2016; Park et al., 2017). Current available treatment for IBS-like symptom focuses on bowel habits and/or visceral pain (Enck et al., 2016; Mao et al., 2017), which seem less effective than expected. Therefore, the therapeutic concept targeting the brain-gut axis is becoming increasingly worthy of attention (Ford et al., 2017).

Resveratrol is a natural polyphenol present in red wine, grape skin and Japanese Knotweed (Nabavi et al., 2017). It has many biological activities including anti-inflammatory, anti-oxidant, anti-neoplastic and neuroprotective (Martín et al., 2006; Lozano-Pérez et al., 2014) effects. Increasing evidence indicates that the antidepressant- and anxiolytic-like properties of resveratrol are found in many animal models of disease, which are related to increases in serotonergic and noradrenergic neurotransmitters (5-hydroxytryptamine, 5-HT and NA) in the brain (Nabavi et al., 2017). Our previous study suggested that antidepressant-like effect of resveratrol was involved in improvement of 5-HT function and its downstream signaling in the hippocampus (Xu et al., 2010). Indeed, nearly 90% of 5-HT is released and stored by enteroendocrine cells in the gastrointestinal tract, which has been proved to participate in regulating gastrointestinal motility (Enck et al., 2016). Therefore, we are the first to determine whether resveratrol could improve IBS-like behaviors, i.e., the central nervous disorders (depression and anxiety) and the peripheral dysfunction (visceral sensitivity and intestinal motility dysfunction), and how resveratrol regulates brain-gut axis by regulating 5-HT-dependent pathway.

In the present study, rats were subjected to the chronic acute combining stress (CACS) to induce depression- and anxiety-like behaviors, intestinal motility alteration and visceral hypersensitivity, which mimicked IBS-like behavior. Treatment with resveratrol before CACS significantly ameliorated the above mentioned central nervous and peripheral symptoms, which were related to its different regulation of 5-HT contents in the brain and the intestine. However, these effects were prevented by 5-HT_1A_ receptor antagonist NAN-190 hydrobromide (NAN-190), which indicate the involvement of 5-HT_1A_ related signaling in treatment of brain-gut axis dysfunction in IBS-like animal models.

## Materials and Methods

### Animals

Male Sprague-Dawley (SD) rats weighing 180–220 g were purchased from SLAC Laboratory Animal Center (Shanghai, China) and acclimatized to the laboratory at least 7 days before the beginning of the experiments. Animals were housed two per cage and had free access to water and food while maintaining a normal 12 h/12 h light/dark cycle before the CACS procedures. Ambient temperature and relative humidity were maintained at 24 ± 1°C and 45 ± 5%. The procedures in this experiment were performed in accordance with the National Institutes of Health Guide for Care and Use of Laboratory Animals (revised 2011) and was approved by the Institutional Animal Care and Use Committee of Wenzhou Medical University.

### Drugs and Treatment

Resveratrol, fluoxetine, diazepam, (±)-8-Hydroxy-2-(dipropylamino) tetralin hydrobromide (8-OH-DPAT, a 5-HT_1A_ receptor agonist), 5-HT and 5-hydroxyindoleacetic acid (5-HIAA) were purchased from Sigma-Aldrich (St. Louis, MO, USA). NAN-190 hydrobromide (NAN-190, a 5-HT_1A_ receptor antagonist) was obtained from Tocris Bioscience (Ellisville, MO, USA).

All drugs were dissolved in saline and diluted to desired concentration on the day of testing except resveratrol that was dissolved in 0.5% sodium carboxymethyl cellulose (CMC-Na). Resveratrol was administered by gavage (i.g.) at doses of 10, 20 and 40 mg/kg with a volume of 5 ml/kg body weight, 50 min before CACS procedure. Fluoxetine (10 mg/kg), diazepam (1 mg/kg), 8-OH-DPAT (0.5 mg/kg) and NAN-190 hydrobromide (0.1 mg/kg) were injected intraperitoneally (i.p.) in a volume of 1 ml/kg. Fluoxetine and diazepam were administered 30 min before CACS procedure. For co-administered with resveratrol, the 5-HT_1A_ receptor agonist and antagonist (8-OH-DPAT and NAN-190) were injected 15 min before resveratrol administration. All the behavioral tests started 24 h after last drug treatment (Figure 1B).

### Chronic Acute Combing Stress (CACS) Procedure

A CACS procedure was performed in rats as described in our previous and other laboratory’s work (Zou et al., 2008; Yu et al., 2015) with minor modifications. Briefly, the CACS-treated rats received chronic unpredictable stress randomly for 22 consecutive days, including food deprivation for 24 h, water deprivation for 24 h, swimming in 4°C cold water for 4 min, tail pinch for 3 min, overnight illumination, and housing in a wet cage for 6 h. On day 22, all rats except the control group received 3 h of acute restraint stress as shown in Figure 1.

### Behavioral Tests

#### Forced Swimming Test (FST)

The forced swimming test (FST) is one of the most commonly used animal models, which assesses the efficacy of antidepressant treatment. The FST consisted of two sessions, i.e., pre-test (on day 23) and formal test (on day 24), which were carried out over two consecutive days (Porsolt et al., 1977). The formal test session was performed 24 h after pre-test. Rats were placed individually into the cylinders (height 45 cm, diameter 25 cm) containing 30 cm of water (22–25°C) and stayed there for 15 min while 5 min in test session. The investigator who was blind to this study recorded the immobility time over the test section. An animal was judged immobile when it was floating passively, performing only slow movements in order to keep its head above the water.

#### Marble-Burying Task (MBT)

The marble-burying task (MBT) was conducted to evaluate anxiety-like behaviors of rats. Animals were placed individually in a plastic chamber (45 × 30 × 40 cm) containing corncob pad (5 cm in depth) and 9 green marbles (2.3 cm in diameter) arranged in three evenly spaced rows and located on top of the corncob pad. After a 10-min exposure to the marbles, rats were removed and the buried marbles were counted. The investigator who was blind to this study recorded the number of marbles buried (at least one-half covered with corncob; Schneider and Popik, 2007).

#### Intestinal Tract Motility Test

The number of fecal output used as an indirect measure of ITM was assessed as described previously (Yu et al., 2015). Rats were placed individually into plexiglass restraining cylinders (length 18 cm, diameter 6 cm) for 1 h. The number of pellets expelled by each animal during 1-h restraint period was counted.

#### Abdominal Withdrawal Reflex Test (AWR)

The visceral sensitivity to colorectal distention (CRD) was evaluated by measuring the abdominal withdrawal reflex (AWR) as described previously (Al-Chaer et al., 2000; Winston et al., 2007). Briefly, the rats were freely access to water, but with food deprivation 24 h before the procedure, and then they were lightly anesthetized with 2% isoflurane, a DIY balloon (5 cm of length; constructed from latex glove finger) attached to a catheter (2 mm of diameter) was inserted 6 cm into the descending colon and rectum until the catheter was positioned to the anus (1 cm distal from the end of the balloon) and held in place by taping the tubing to the tail. After that, rats were placed in Lucite cubicles (20 × 8 × 8 cm) and allowed 20 min (after awaking) to acclimate to the environment before testing. Distention pressures (20, 40, 60 and 80 mmHg) were given for 20 s with a 4-min interval between different pressures. Behavioral responses to CRD were measured by visual observation of the AWR and the assignment of an AWR score as Table 1.

**Table 1:** The effects of resveratrol on abdominal withdrawal reflex (AWR) score.

Score	Behavioral responses to CRD
0	No behavioral response to CRD
1	Brief head movement followed by immobility
2	Contraction of abdominal muscles
3	Lifting of abdomen
4	Body arching and lifting of pelvic structures

### Biochemical Analyses

#### Determination of Brain and Colonic 5-HT and Its Metabolite Levels

After the end of the behavioral experiment, the rats were decapitated after anesthetized with 10% chloral hydrate, then the hippocampus, ileum and colon were collected and weighed before being stored at −80°C until analysis. The content of 5-HT and 5-HIAA were detected by Agilent 1260 HPLC system with electrochemical detector from Antec. The pre-treatment method of the samples was carried out according to our previously established protocol (Chen et al., 2015). Supernatants of samples (20 μl) were separated on a Diamonsil C18 column (150 × 4.6 mm, 5 μm particle size) with the temperature setting at 35°C. The mobile phase consisted of 100 mM NaH_2_PO_4_, 0.74 mM sodium octane sulfonate, 0.027 mM EDTA and 15% methanol (adjusted pH to 3.05 with acetic acid, flow rate 1.0 ml/min). The detector connected to a glass carbon electrode (Ag/AgCl reference) was set at 0.52 V within output range of 10 nA. The tissue levels of monoamine were expressed in terms of nanograms per gram of tissue.

### Immunoblot Analyses

The tissues (hippocampus, ileum and colon) were homogenized in RIPA lysis buffer containing protease and phosphatase inhibitors (PMSF:RIPA = 1:100), and clarified twice by centrifugation at 12,000 rpm for 20 min at 4°C. The protein content of supernatants was quantified using Enhanced BCA Protein Assay Kit (Beyotime; catalog no. P0010). The proteins were diluted with RIPA lysis buffer containing loading buffer (5×) into final concentration of 4 μg/μl, then each sample in the volume of 15 μl was subjected to Western blot analysis (Wang et al., 2017). Antibodies included anti-PKA (Abcam; Catalog No. ab76238; 1:50,000), anti-p-cAMP-response element binding protein (anti-p-CREB; Millipore; Catalog No. 06-519; 1:1,000), anti-CREB (Millipore; Catalog No. 06-863; 1:500), anti-brain derived neurotrophic factor (anti-BDNF; Abcam; Catalog No. ab216443; 1:2,500), anti-GAPDH (Bio-world; Catalog No. AP0063; 1:5,000), anti-β-actin (Bio-world; Catalog No. AP0060; 1:5,000) and goat anti-rabbit IgG (H + L; Multi Sciences; Catalog No. GAR007; 1:15,000). The blots were quantified using Quantity One software version 4.6.2.

### Data Analysis

The values were presented as mean ± standard error (mean ± SEM). Data were analyzed with independent samples *t*-test, one-way analysis of variance (ANVOA) and followed by Dunnett’s multiple comparison *post hoc* test when appropriate, using SPSS version 18.0. Differences with *P* < 0.05 were considered significant.

## Results

### CACS-Induced Depression/Anxiety-Like Behavior and Intestinal Dysfunction Were Prevented by Resveratrol

**Figure 2:**
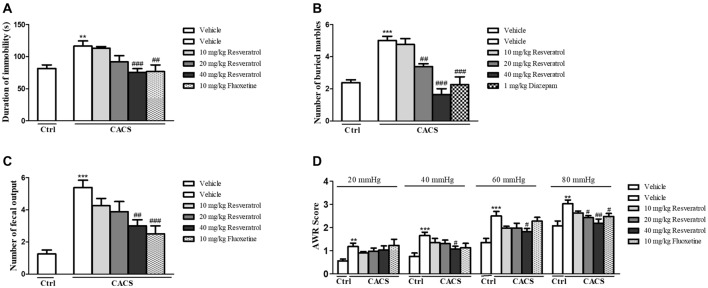
The effects of resveratrol on central nervous and peripheral systems in the forced swimming **(A)**, marble burying **(B)**, the number of fecal outputs in 1-h restraint period **(C)** and AWR score **(D)**. Values are expressed as mean ± SEM (*n* = 6–8, per group), **P < 0.01, ***P < 0.001 vs. vehicle-treated control group (“Veh + Ctrl” group); ^#^*P* < 0.05, ^##^*P* < 0.01, ^###^*P* < 0.001 vs. vehicle-treated CACS group (“Veh + CACS” group).

The experiment procedure was shown in Figure 1B. The role of resveratrol in regulation of depressant-like behavior in CACS rats was assessed by the FST as shown in Figure 2A. CACS induced significant increases in immobility time of rats in FST (*P* < 0.01), which was prevented by resveratrol in a dose dependent manner (*F*_(3,28)_ = 7.36, *P* < 0.001). The effect was similar to that of classical antidepressant fluoxetine (*P* < 0.01). The subsequent study suggested that rats exposed to CACS exhibited a significant increase in the number of marbles buried in MBT as shown in Figure 2B (*P* < 0.001). However, this increase was reversed by treatment with resveratrol at 10, 20 and 40 mg/kg (*F*_(3,28)_ = 25.31, *P* < 0.001) or the positive drug diazepam at 1 mg/kg (*P* < 0.001). The results suggested that resveratrol exhibits antidepressant- and anxiolytic-like effects in the rat model of CACS.

We examined the number of fecal outputs in rats during 1h-restraint period on day 25 to measure the effect of resveratrol on the ITM dysfunction. As shown in Figure 2C, the fecal pellet outputs in the vehicle-treated CACS group increased nearly two times as compared to the vehicle-treated control group (*P* < 0.001). However, resveratrol dose-dependently blocked the increased fecal pellet outputs significantly (*F*_(3,28)_ = 4.00, *P* < 0.05), which was similar to that observed in the positive drug fluoxetine (*P* < 0.001). We also determined the effect of resveratrol on the visceral pain response by AWR, which is often used as a semi-quantitative visceral pain assessment method. As shown in Table 1 and Figure 2D, the AWR score in response to CRD in the vehicle-treated CACS group was significantly higher than respective control groups at distension pressures of 20, 40, 60 and 80 mmHg (*P* < 0.01, *P* < 0.001, *P* < 0.001 and *P* < 0.01, respectively). The increased AWR score was reversed by treatment animals with resveratrol at distention pressures of 40 mmHg (*F*_(3,28)_ = 2.433, *P* < 0.05), 60 mmHg (*F*_(3,28)_ = 3.259, *P* < 0.05) and 80 mmHg (*F*_(3,28)_ = 6.595, *P* < 0.05), respectively. Fluoxetine-treated groups also decreased the AWR at the distension pressure of 80 mmHg. These results suggested that resveratrol rescues the CACS-induced IBS-like intestinal dysfunction, i.e., intestinal motility disorder and visceral hypersensitivity.

### Resveratrol Reversed 5-HT and Its Metabolites Abnormalities in the Hippocampus, Ileum and Colon of IBS Rats

As shown in Table 2, 5-HT level was markedly decreased after CACS in the hippocampus (*P* < 0.01); while the ratio of 5-HIAA/5-HT was significantly increased (*P* < 0.01). Chronic treatment with resveratrol significantly increased 5-HT level in the hippocampus (*P* < 0.05) and decreased the ratio of 5-HIAA/5-HT at 40 mg/kg (both *P* < 0.05) when compared to those vehicle-treated CACS rats. These effects were also observed by treatment with fluoxetine (10 mg/kg) in CACS rats. Surprisingly, 5-HT levels were significantly increased in the ileum and colon after CACS (*P* < 0.05), while the ratio of 5-HIAA/5-HT was significantly decreased both in the ileum and colon (both *P* < 0.05) when compared to the vehicle-treated control rats, which were opposite to the changes in the hippocampus. However, resveratrol reversed 5-HT abnormalities in the ileum and colon of CACS rats, as evidenced by decreased 5-HT levels after treatment with resveratrol at dose of 40 mg/kg both in the ileum and colon (*P* < 0.05 and *P* < 0.01) and a significant increase in the 5-HIAA/5-HT ratio in the colon (*P* < 0.05). These resveratrol-induced changes in 5-HT contents in both the ileum and colon and the related metabolic rate were similar to those of fluoxetine.

**Table 2:** The effects of resveratrol on 5-HT and 5-HIAA contents in the brain and intestine of irritable bowel syndrome (IBS) rats.

Group		Concentration (ng/g)		
		5-HT	5-HIAA	5-HIAA/5-HT
Hippocampus	Control + vehicle	228.90 ± 19.54	68.46 ± 7.17	0.30 ± 0.37
	CACS + vehicle	147.51 ± 12.70**	92.80 ± 3.57*	0.63 ± 0.28**
	CACS + RES 10	146.00 ± 9.87	80.83 ± 6.08	0.55 ± 0.62
	CACS + RES 20	182.90 ± 10.60	77.82 ± 4.41	0.43 ± 0.42^#^
	CACS + RES 40	196.55 ± 14.78^#^	76.84 ± 2.54^#^	0.39 ± 0.17^#^
	CACS + FLU 10	197.30 ± 13.74^#^	80.85 ± 5.67	0.41 ± 0.41
Ileum	Control + vehicle	1209.05 ± 144.78	220.85 ± 12.67	0.20 ± 0.04
	CACS + vehicle	1632.35 ± 109.99*	163.26 ± 18.53*	0.10 ± 0.02*
	CACS + RES 10	1436.12 ± 115.19	167.15 ± 21.54	0.12 ± 0.02
	CACS + RES 20	1289.35 ± 112.43	178.50 ± 23.26	0.15 ± 0.02
	CACS + RES 40	1173.85 ± 163.23^#^	170.07 ± 27.27	0.17 ± 0.04
	CACS + FLU 10	1204.11 ± 97.05^#^	178.20 ± 24.52	0.17 ± 0.03
Colon	Control + vehicle	2231.43 ± 312.65	148.57 ± 10.80	0.07 ± 0.01
	CACS + vehicle	3462.86 ± 253.12*	116.73 ± 22.39	0.04 ± 0.01*
	CACS + RES 10	2822.61 ± 309.11	106.12 ± 8.01	0.04 ± 0.00
	CACS + RES 20	2591.75 ± 383.80	125.47 ± 21.18	0.05 ± 0.01
	CACS + RES 40	2131.02 ± 309.48^##^	143.25 ± 23.49	0.07 ± 0.02^#^
	CACS + FLU 10	2514.13 ± 117.03^#^	114.91 ± 16.14	0.05 ± 0.01

RES, resveratrol; FLU, fluoxetine. Values were expressed as mean ± SEM of six rats. *P < 0.05 and **P < 0.01, compared to vehicle treated control rats. ^#^P < 0.05 and ^##^P < 0.01, compared to vehicle treated chronic acute combining stress (CACS) rats.

### The Antidepressant- and Anxiolytic-Like Effects of Resveratrol Were Involved in Recovery of 5-HT_1A_ Receptor Functions in Rat Model of CACS

**Figure 3:**
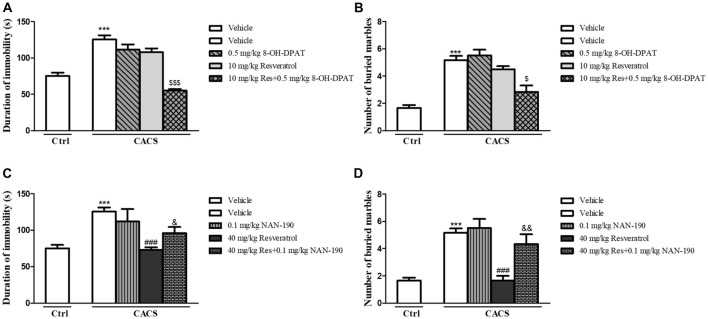
The antidepressant- **(A,C)** and anxiolytic-like **(B,D)** effects of resveratrol in irritable bowel syndrome (IBS) rats were involved in 5-HT_1A_ receptor. Values are expressed as mean ± SEM (*n* = 6–8, per group), ***P < 0.001 vs. vehicle-treated control group (“Veh + Ctrl” group); ^###^*P* < 0.001 vs. vehicle-treated CACS group (“Veh + CACS” group); ^$^*P* < 0.05, ^$$$^*P* < 0.001 vs. 10 mg/kg resveratrol-treated CACS group (“RES 10 + CACS” group); ^&^*P* < 0.05, ^&&^*P* < 0.01 vs. 40 mg/kg resveratrol-treated CACS group (“RES 40 + CACS” group).

In order to determine whether the effects of resveratrol on depression- and anxiety-like behavior were related to 5-HT and its receptor’s function, the antidepressant- and anxiolytic-like effects of sub-threshold dose of resveratrol (10 mg/kg) combined with low dose of 5-HT_1A_ receptor agonist 8-OH-DPAT (0.5 mg/kg) were investigated. Although both of 8-OH-DPAT and resveratrol at low doses did not induce any effects in the FST (Figure 3A) and MBT (Figure 3B) respectively, the combination of these two compounds reduced the immobility time and the number of buried marbles (FST, *P* < 0.001; MBT, *P* < 0.05). The further studies suggested that 5-HT_1A_ receptor antagonist NAN-190 at 0.1 mg/kg prevented the effects of high dose of resveratrol at 40 mg/kg on immobility time and the number of buried marbles in the FST (Figure 3C) and MBT (Figure 3D; FST, *P* < 0.05; MBT, *P* < 0.01). These effects supported that 5-HT_1A_ receptor involves the antidepressant- and anxiolytic-like effects of resveratrol in both FST and MBT.

### The Effects of Resveratrol on CACS-Induced Intestinal Motility Abnormality and Visceral Hypersensitivity Were Involved in Regulation of 5-HT_1A_ Receptor Function

**Figure 4:**
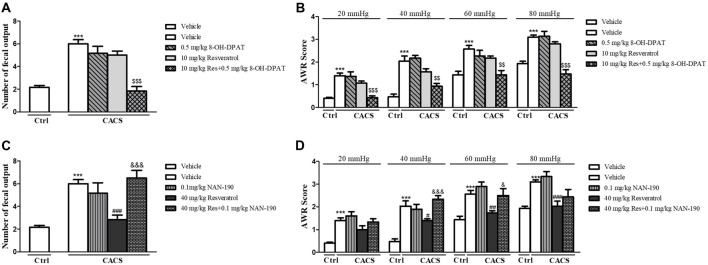
The effects of resveratrol on CACS-induced intestinal motility disorder **(A,C)** and visceral hypersensitivity **(B,D)** were involved in regulation of 5-HT_1A_ receptor function. Values are expressed as mean ± SEM (*n* = 6–8, per group), ***P < 0.001 vs. “Veh + Ctrl” group; ^#^*P* < 0.05, ^##^*P* < 0.01, ^###^*P* < 0.001 vs. “Veh + CACS” group; ^$$^*P* < 0.01, ^$$$^*P* < 0.001 vs. “RES 10 + CACS” group; ^&^*P* < 0.05, ^&&&^*P* < 0.001 vs. “RES 40 + CACS” group.

We further determined the involvement of 5-HT_1A_ in effects of resveratrol on intestinal dysfunction. As shown in Figure 4, the low dose of 5-HT_1A_ receptor agonist 8-OH-DPAT potentiated the effects of sub-threshold dose of resveratrol (10 mg/kg) on the number of fecal outputs (Figure 4A, *P* < 0.001), and AWR at 20, 40, 60 and 80 mmHg (Figure 4B, *P* < 0.001, *P* < 0.01, *P* < 0.01 and* P* < 0.001, respectively) in the ITM and AWR tests. However, the 5-HT_1A_ antagonist NAN-190 prevented the effects of high dose of resveratrol at 40 mg/kg on both the number of fecal outputs (Figure 4C, *P* < 0.001), and AWR at 40 and 60 mmHg (Figure 4D, *P* < 0.001 and *P* < 0.01).

### The Normalized PKA, pCREB and BDNF Levels in the Hippocampus by Resveratrol Were Involved in Regulation of 5-HT_1A_ Receptor Function in the CACS Rats

**Figure 5:**
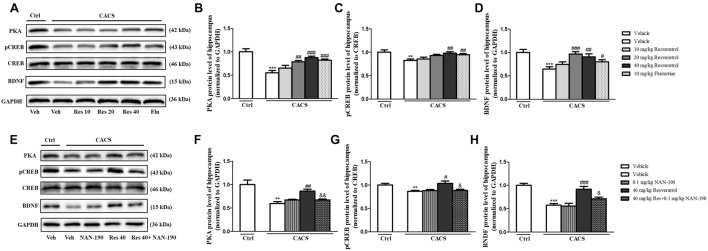
The effects of resveratrol on PKA, pCREB/CREB and brain derived neurotrophic factor (BDNF) levels in the hippocampus. **(A)** Photomicrographs of representative immune-blotting bands. Lane 1: “Veh + Ctrl” group, lane 2: “Veh + CACS” group, lane 3: “RES 10 + CACS” group, lane 4: “RES 20 + CACS” group, lane 5: “RES 40 + CACS” group and lane 6: “FLU 10 + CACS” group. **(B–D)** PKA, the ratio of pCREB to CREB and BDNF expression in the hippocampus. **(E)** Photomicrographs of representative immune-blotting bands. Lane 1: “Veh + Ctrl” group, lane 2: “Veh + CACS” group, lane 3: “NAN-190 + CACS” group, lane 4: “RES 40 + CACS” group and lane 5: “RES 40 + NAN-190 + CACS” group. **(F–H)** PKA, the ratio of pCREB to CREB and BDNF expression in the hippocampus. Values are expressed as mean ± SEM (*n* = 6–8, per group), **P < 0.01, ***P < 0.001 vs. “Veh + Ctrl” group; ^#^*P* < 0.05, ^##^*P* < 0.01, ^###^*P* < 0.001 vs. “Veh + CACS” group; ^ &^*P* < 0.05, ^&&^*P* < 0.01 vs. “RES 40 + CACS” group.

As shown in Figure 5A, the immune-blot analyses demonstrated that PKA, the ratio of pCREB to total CREB (pCREB/CREB) and BDNF expression were decreased in the hippocampus when rats were subjected to CACS for 22 days (PKA, *P* < 0.001; pCREB/CREB, *P* < 0.01 and BDNF, *P* < 0.001, Figures 5B–D). Treatment with resveratrol significantly increased PKA expression (*P* < 0.01 and *P* < 0.001), the ratio of pCREB/CREB (*P* < 0.01) and BDNF level (*P* < 0.001 and *P* < 0.01) at 20 or 40 mg/kg. The reduction of these proteins induced by CACS was also ameliorated by treatment with fluoxetine (PKA, *P* < 0.001; pCREB/CREB, *P* < 0.01 and BDNF, *P* < 0.05) when compared to the vehicle-treated CACS groups, respectively.

To determine whether 5-HT_1A_ receptor mediates the anti-IBS-like effects of resveratrol, we observed whether pretreatment of 5-HT_1A_ antagonist NAN-190 (0.1 mg/kg) blocked the effects of resveratrol on PKA, pCREB/CREB and BDNF expression in the hippocampus. As shown in Figures 5E–H, pretreatment of NAN-190 could inhibit the effects of high dose of resveratrol (40 mg/kg) on the above-mentioned proteins expression, i.e., significantly reduced PKA (*P* < 0.01), the ratio of pCREB/CREB (*P* < 0.05) and BDNF expression (*P* < 0.05) in the hippocamps when compared to the CACS rats that received resveratrol at dose of 40 mg/kg. Meanwhile, treatment with NAN-190 itself did not affect these three proteins expression in the hippocampus.

### Resveratrol-Induced Increases in PKA, pCREB and BDNF Levels in the Ileum of CACS Rats Were Blocked by the 5-HT_1A_ Receptor Antagonist NAN-190

**Figure 6:**
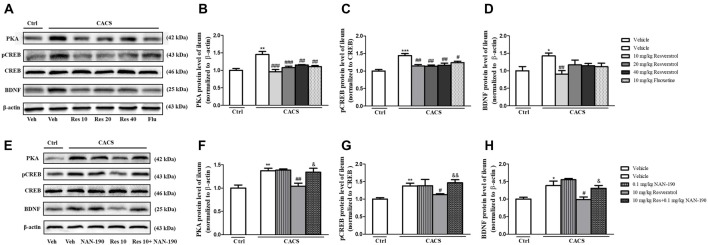
The effects of resveratrol on PKA, pCREB/CREB and BDNF expression in the ileum. **(A)** Photomicrographs of representative immune-blotting bands. Lane 1: “Veh + Ctrl” group, lane 2: “Veh + CACS” group, lane 3: “RES 10 + CACS” group, lane 4: “RES 20 + CACS” group, lane 5: “RES 40 + CACS” group and lane 6: “FLU 10 + CACS” group. **(B–D)** PKA, the ratio of pCREB to CREB and BDNF expression in the ileum. **(E)** Photomicrographs of representative immune-blotting bands. Lane 1: “Veh + Ctrl” group, lane 2: “Veh + CACS” group, lane 3: “NAN-190 + CACS” group, lane 4: “RES 10 + CACS” group and lane 5: “RES 10 + NAN-190 + CACS” group. **(F–H)** PKA, the ratio of pCREB to CREB and BDNF expression in the ileum. Values are expressed as mean ± SEM (*n* = 6–8, per group), *P < 0.05, **P < 0.01, ***P < 0.001 vs. “Veh + Ctrl” group; ^#^*P* < 0.05, ^##^*P* < 0.01, ^###^*P* < 0.001 vs. “Veh + CACS” group; ^&^*P* < 0.05, ^&&^*P* < 0.01 vs. “RES 10 + CACS” group.

PKA, BDNF and pCREB/CREB levels were significantly increased in the ileum of the vehicle-treated CACS groups, as compared to those of vehicle-treated control groups (Figures 6A–D; PKA, *P* < 0.01; pCREB/CREB, *P* < 0.001 and BDNF, *P* < 0.05, respectively). Treatment with resveratrol from 10 to 40 mg/kg significantly decreased the PKA, the ratio of pCREB/CREB and BDNF levels in the ileum of CACS rats (PKA: *F*_(3,20)_ = 12.41, *P* < 0.001; pCREB/CREB, *F*_(3,22)_ = 7.625, *P* < 0.01; BDNF: *F*_(3,20)_ = 4.502, *P* < 0.05). Surprisingly, it seemed that 10 mg/kg resveratrol was sufficient to rescue the abnormalities of all three proteins expression in the ileum of CACS rats. Fluoxetine also decreased PKA (*P* < 0.01) and pCREB/CREB (*P* < 0.05) expression in the ileum, as compared to vehicle-treated CACS groups. The results in Figures 6E–H showed that pretreatment of NAN-190 (0.1 mg/kg) could prevent the effects of resveratrol at 10 mg/kg by significantly increasing PKA (*P* < 0.05), the ratio of pCREB/CREB (*P* < 0.01) and BDNF expression (*P* < 0.05) in the ileum. However, NAN-190 (0.1 mg/kg) used alone did not affect these proteins expression in the ileum of CACS rats.

**Figure 7:**
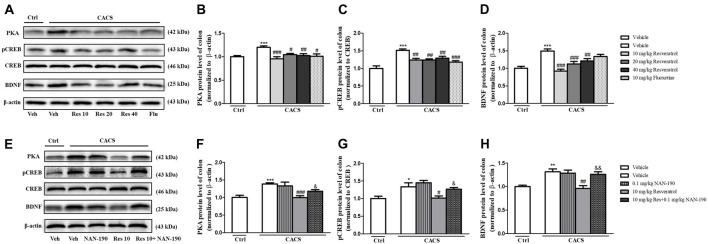
The effects of resveratrol on PKA, pCREB/CREB and BDNF expression in the colon. **(A)** Photomicrographs of representative immune-blotting bands. Lane 1: “Veh + Ctrl” group, lane 2: “Veh + CACS” group, lane 3: “RES 10 + CACS” group, lane 4: “RES 20 + CACS” group, lane 5: “RES 40 + CACS” group and lane 6: “FLU 10 + CACS” group. **(B–D)** PKA, the ratio of pCREB to CREB and BDNF expression in the colon. **(E)** Photomicrographs of representative immune-blotting bands. Lane 1: “Veh + Ctrl” group, lane 2: “Veh + CACS” group, lane 3: “NAN-190 + CACS” group, lanes 4: “RES 10 + CACS” group and lane 5: “RES 10 + NAN-190 + CACS” group. **(F–H)** PKA, the ratio of pCREB to CREB and BDNF expression in the colon. Values are expressed as mean ± SEM (*n* = 6–8, per group), *P < 0.05, **P < 0.01, ***P < 0.001 vs. “Veh + Ctrl” group; ^#^*P* < 0.05, ^##^*P* < 0.01, ^###^*P* < 0.001 vs. “Veh + CACS” group; ^&^*P* < 0.05, ^&&^*P* < 0.01 vs. “RES 10 + CACS” group.

Similar results were found in the colon as shown in Figures 7A–D, the significant increases in PKA (*P* < 0.001), pCREB/CREB (*P* < 0.001) and BDNF (*P* < 0.001) in the colon of vehicle-treated CACS groups were also found. Treatment with resveratrol from 10 to 40 mg/kg decreased PKA (*F*_(3,20)_ = 7.89, *P* < 0.01), pCREB/CREB (*F*_(3,22)_ = 7.612, *P* < 0.01) and BDNF (*F*_(3,28)_ = 14.33, *P* < 0.001) levels significantly, which were similar to those of fluoxetine. The data demonstrated that resveratrol at 10 mg/kg was enough to reduce these three proteins expression in the colon of CACS rats, which were consistent with the results from the ileum. The further study seen in the Figures 7E–H suggested that the 5-HT_1A_ antagonist NAN-190 (0.1 mg/kg) significantly reversed the effects of resveratrol at 10 mg/kg on the above mentioned three parameters, which were similar to those in the ileum.

## Discussion

The present study suggested that rats subjected to 22 days CACS induced the abnormalities in the brain-gut axis, i.e., emotional disorders in the CNS and intestinal dysfunction in the peripheral system. Chronic treatment with resveratrol for 22 days before CACS procedure exhibited dual effects in the central nervous and intestinal systems, as evidenced by the significant antidepressant- and anxiolytic-like effects, rescuing intestinal motility disorder and visceral hypersensitivity. The further study suggested that the dual effects of resveratrol on CNS and peripheral systems were potentiated by pretreatment with low dose of 5-HT_1A_ agonist 8-OH-DPAT, but were reversed by the 5-HT_1A_ antagonist NAN-190. Interestingly, the results suggested that resveratrol at the dose of 40 mg/kg achieved the maximal protective effects against CACS-induced CNS disorders. However, resveratrol at dose of 10 mg/kg seemed enough to rescue molecular signaling dysfunction in the peripheral system. Considering that our pilot study indicated that the drug concentrations were different in the brain and plasma (brain: 0.0125–0.1141 nmol/ml; plasma: 0.3204–1.9120 nmol/ml), 30 min after resveratrol was administered at doses of 10, 20, 40, and 80 mg/kg *via* gavage (Xu et al., 2010), it is possible that higher concentration of resveratrol is needed to achieve maximal effects in the brain. Our results suggested that resveratrol protects rats against CACS attack by dual regulation of 5-HT_1A_ dependent signaling in the CNS and the peripheral system though the doses of resveratrol may be different in these two systems.

IBS is considered as an intestinal dysfunction with abdominal pain and discomfort, and changes in bowel habits, which also comorbid with emotional disorders, such as depression and anxiety. Although the underlying pathogenesis of IBS has not yet been fully elucidated, etiological factors including the gastrointestinal motility dysfunction, visceral hypersensitivity, intestinal infections and brain-gut interactions are involved (Enck et al., 2016). Recent studies indicated that IBS-related pain and colorectal hypersensitivity could be induced by an increased primary sensory afferent derived from the colorectum (Cervero and Laird, 2004; Christianson et al., 2009; Feng et al., 2012). Moreover, long-term abnormalities in the brain that are associated with emotional process in IBS patients have been brought up recently because the brain regions could be messed up by increased sensory inputs from the peripheral system (Enck et al., 2016). This pathological mechanism of IBS results in some animal models developed in previous studies that were only focusing on peripheral sensory mechanism, such as phasic or repetitive CRD, and injection of inflammatory chemicals into the colon. Stress-induced IBS models, e.g., restraint stress and water deprivation stress, could largely mimic IBS symptoms from CNS disorders to intestinal motility and visceral perception disorders (Zou et al., 2008; Greenwood and Fleshner, 2011). In the present study, rats exposed to 22-days’ CACS showed depression- and anxiety-like behaviors that were measured in the forced swimming and marble-burying tests. These CACS rats also showed visceral hypersensitivity and altered intestinal motility in behavior. These abnormalities of behavior in the CNS and peripheral system were similar to the psychiatric and somatic disorders in individuals suffering from IBS. Considering that resveratrol plays an important role in the treatment of stress-related depression- and anxiety-like behavior in our previous studies (Xu et al., 2010; Yu et al., 2013), we expected that resveratrol exhibited dual effects on both affective and somatic disorders induced by CACS. The present results suggested that these behavioral abnormalities related to brain-gut axis dysfunction induced by CACS were reversed by resveratrol, which not only ameliorated depression- and anxiety-like behavior, but also improved intestinal hypersensitivity and dysfunction as found in the AWR score and ITM behavioral tests.

5-HT is widely present in the central nervous and gastrointestinal systems in which it plays an important role in the brain-gut network. For example, 5-HT system dysfunction results in diarrhea-predominant irritable bowel syndrome (D-IBS), in which the gastrointestinal motility and viscera sensitivity are increased (Siegert and Nieber, 2010; Grenham et al., 2011). The 5-HT_1A_ receptor is one of the most important 5-HT receptors related to depression (Pérez-Cáceres et al., 2013), anxiety (Podona et al., 1994), and stress-induced dyspeptic ulcers and anxiety (Podona et al., 1994). Therefore, targeting at 5-HT neurotransmitter and its 5-HT_1A_ receptor may be a promising approach for treatment of IBS-like symptoms including psychiatric and gastrointestinal dysfunction. In the present study, we found that the effects of resveratrol on 5-HT and its metabolites in the CNS and peripheral system were opposite, i.e., increased 5-HT level and decreased the metabolic rate in the hippocampus, while decreased 5-HT level and increased the ratio of 5-HIAA to 5-HT were significant in the colon. These results reflected that the negative feedback in the brain-gut axis might be activated for maintaining body’s function under CACS status. The subsequent study showed that low dose of 5-HT_1A_ receptor agonist 8-OH-DPAT potentiated sub-threshold dose of resveratrol’s effects on IBS-like symptoms; while pretreatment with 5-HT_1A_ antagonist NAN-190 prevented such effects, further supporting the involvement of 5-HT and the 5-HT_1A_ receptor in the effects of resveratrol on IBS-like symptoms.

5-HT neurotransmitter promotes cyclic AMP (cAMP) synthesis by activation of 5-HT receptor subtypes, such as 5-HT_1A_, 5-HT_4_ and 5-HT_7_ (Markstein et al., 1999). Altered cAMP signaling is implicated in major depressive disorder and gastrointestinal dysfunction (Cowburn et al., 1994), which has been recognized as a potential target for treatment of IBS-like symptoms. Cycle AMP induces the sensitization of cAMP-dependent protein kinase (PKA), which in turn triggers the downstream cAMP signaling such as CREB. Phosphorylation of CREB regulates transcriptional activity and stimulates BDNF expression, leading to antidepressant, anxiolytic and neuroprotective effects (Reichardt, 2006; Yossifoff et al., 2008). The present study suggested that resveratrol rescued the CACS-induced decreases in PKA, pCREB and BDNF expression in the hippocampus. However, CACS-induced increases in PKA, pCREB and BDNF expression in the ileum and colon were reversed by resveratrol at a relative low dose of 10 mg/kg. The main reason might be involved in the low penetration of resveratrol to blood-brain barrier, which indicate that the higher dose of resveratrol might be necessary to change CNS symptom, i.e., depression- and anxiety-like behaviors. Considering that intestinal system receives messages from both CNS and peripheral system to maintain its normal function, higher dose of resveratrol may be necessary to reverse CACS-induced intestinal dysfunction. This may explain why high dose of resveratrol (40 mg/kg) is still needed for rescuing CACS relevant intestinal motility disorder and visceral hypersensitivity although relative low dose of resveratrol (10 mg/kg) is enough to regulate the molecular signaling in the intestine.

Taking together, resveratrol ameliorated CACS-induced IBS-like symptoms such as depression-, anxiety-like behaviors and gastrointestinal dysfunction. The changes in 5-HT and its metabolic rate and 5-HT_1A_ receptor, as well as PKA, pCREB and BDNF expression after treatment with resveratrol support the fact that resveratrol produces anti-IBS-like effects *via* regulation of 5-HT_1A_ receptor-dependent PKA-CREB-BDNF signaling.

## Author Contributions

YX and J-MS conceived and designed the study, provided critical comments and edited the manuscripts. Y-CY performed experiments on rat including the acquisition, analysis and interpretation of data. JL and MZ performed analysis and interpretation of data on immunoblot analysis and Biochemical analyses. J-CP and YY performed on data collecting. J-BZ drafted and revised the paper. LZ edited the paper. All authors read and approved the final manuscript.

## Conflict of Interest Statement

The authors declare that the research was conducted in the absence of any commercial or financial relationships that could be construed as a potential conflict of interest.
